# Knowledge and use of family planning among men in rural Uganda

**DOI:** 10.1186/s12889-018-6173-3

**Published:** 2018-11-26

**Authors:** Anne Dougherty, Alex Kayongo, Samantha Deans, John Mundaka, Faith Nassali, James Sewanyana, Eric Migadde, Ronald Kiyemba, Estherloy Katali, Sarah Jane Holcombe, Sarah H. Heil, Robert Kalyesubula

**Affiliations:** 1 Department of Obstetrics, Gynecology & Reproductive Sciences, University of Vermont, Burlington, VT USA; 2 Department of Medicine, Makerere College of Health Sciences, Kampala, Uganda; 3 African Community Center for Social Sustainability (ACCESS), Nakaseke, Uganda; 4 Department of Obstetrics and Gynecology, Makerere College of Health Sciences, Kampala, Uganda; 5 Makerere University Business School, Kampala, Uganda; 6 Bixby Center for Population, Health, and Sustainability, University of California, Berkeley, USA; 7 Departments of Psychiatry and Psychological Science, University of Vermont, Burlington, VT USA; 8 Departments of Medicine and Physiology, Makerere College of Health Sciences, P.O Box 7072, Kampala, Uganda

**Keywords:** Family planning, Contraception, Men, Contraceptive knowledge, Uganda, Low resource

## Abstract

**Background:**

Unmet need for family planning exceeds 33% in Uganda. One approach to decreasing unmet need is promoting male involvement in family planning. Male disapproval of use of family planning by their female partners and misconceptions about side effects are barriers to family planning globally and in Uganda in particular. Researchers have conducted a number of qualitative studies in recent years to examine different aspects of family planning among Ugandan men. The present study aimed to quantify men’s knowledge of family planning in rural Uganda to understand how better to involve men in couples’ contraceptive decision-making, particularly in low-resource settings.

**Methods:**

Data were derived from in-person, researcher-administered surveys of men in a rural agrarian district in Uganda (*N* = 178). Participant demographics and knowledge of family planning methods, side effects, and use were queried. Descriptive statistics were used for analysis.

**Results:**

Men were 34 years of age on average (range 18–71) and about half (56%) had a primary school education or less. Ninety-eight percent reported any knowledge of family planning, with 73% of men reporting obtaining information via radio and only 43% from health workers. The most common method known by men was the male condom (72%), but more than half also knew of injections (54%) and pills (52%). Relatively few men reported knowing about the most effective reversible contraceptive methods, intrauterine devices and implants (both 16%). Men identified many common contraceptive side-effects, such as vaginal bleeding (31%), and misconceptions about side effects, such as increased risk of infertility and birth defects, were relatively uncommon (both < 10%). About half of all men reported ever using a family planning method (53%), and 40% reported current use.

**Conclusions:**

This study’s quantitative results build on those of recent qualitative studies and provide information about the types of family planning information men are lacking and avenues for getting this information to them.

**Electronic supplementary material:**

The online version of this article (10.1186/s12889-018-6173-3) contains supplementary material, which is available to authorized users.

## Background

Family planning helps couples and individuals decide if and when to have children. The growing use of family planning worldwide has resulted in reductions in maternal and infant mortality and other adverse outcomes [1–3]. Still, nearly 25% of women in Sub-Saharan Africa have an unmet need for family planning [4] and rates exceed 33% in a few countries like Uganda.

In September 2017, Uganda held a 2nd National Family Planning Conference where the Minister of Health emphasized the central goals of the Uganda Costed Implementation Plan for Family Planning (2015–2020) to reduce unmet need for family planning to 10% by 2020 and increase contraceptive use by married women to 50% [5]. One of the approaches put forth at the conference for decreasing unmet need was promoting male involvement in family planning [6]. Data from the most recent Uganda Demographic and Health Survey (UDHS) indicate that men are involved: 62% of married Ugandan women reported that contraceptive decisions are made jointly with their male partners and 7% state that decisions are made exclusively by their male partners [6]. However, unmet need remains among the highest in the world, suggesting that many men are deciding that their partners should not use contraception, a conclusion supported by other recent studies [7, 8].

In an effort to better understand male knowledge and attitudes concerning family planning, researchers have conducted a number of qualitative studies in recent years to examine where Ugandan men obtain knowledge about family planning, their perceptions of the safety and efficacy of different contraceptive methods, and other related topics [7, 9–11]. These studies have often focused on men living in rural areas, as unmet need is reliably higher in rural compared to urban areas [12, 13]. One recent study involved interviews with 41 men living in rural and peri-urban areas in Rakai, Uganda [11]. Men reported that they acquired most information about family planning from their partners, their peers, or hearsay, with few men reporting having received information about contraceptives from mass media campaigns or health providers. Regarding contraceptive safety and efficacy, men expressed concerns about intrauterine devices (IUDs) migrating inside a woman’s body and thereby losing their efficacy, as well as the belief that condoms can get stuck in a woman’s body, possibly resulting in her death. Men also reported a fear that women who used contraception would become infertile and suffer from side effects like excessive menstrual bleeding and weight changes and that the children of women who used contraception would have abnormalities and deformities. These results are very consistent with other qualitative studies conducted with this population in recent years, including observations that males were unlikely to receive contraceptive information from health workers which may account for false beliefs and fears about side effects of modern contraceptive methods [7, 9, 10].

While qualitative studies are very useful in identifying potential areas to target, quantitative studies are also necessary to identify how common different misperceptions are. This is especially important in low resource settings where time and money need to be thoughtfully allocated for maximum benefit. Thus, the present study sought to extend the results of recent qualitative studies by examining and reporting quantitative data on knowledge and beliefs about contraception collected from men living in Nakaseke District, a rural setting approximately 65 km outside of Kampala, the capital of Uganda.

## Methods

Data for this analysis were derived from a larger cross-sectional, mixed methods community-based study conducted in Nakaseke District in rural central Uganda with both men and women. Nakaseke District occupies 3477 km^2^ with a population of 191,000. Fifty-nine percent of residents are literate, and the majority are farmers using traditional methods.

Research personnel were trained prior to the start of the study in a lecture and role playing format. Participant eligibility was based on place of residence and age. Informed consent was obtained prior to survey completion. The survey of knowledge, use, and accessing of family planning was administered to a convenience sample of men (age 15 or older) and women (age 15–49) in each of six randomly selected villages within the district (Nakaseke town A, Kiziba, Namirali, Kito, Kakoola and Lusanja). The survey was written in English, translated into Luganda (the local language), back translated into English to ensure fidelity and ultimately administered in Luganda. Demographic information was recorded but data were otherwise de-identified. Focus group discussions concerning availability of family planning services and a health care provider survey were also undertaken; results from these efforts are not reported here. In addition, given there is little information about male knowledge, use, and accessing of family planning, the present study reports on data collected from men; results from women are not reported here.

To assess knowledge of family planning practices, broadly speaking, men were asked if they had heard about family planning before (yes/no). If yes, they were asked where they had heard about it. Men were then asked if they knew of any family planning methods (yes/no). If yes, they were asked to name the family planning method(s) that they knew of. They were then asked if they had heard of any side effects of some family planning methods (yes/no). If yes, they were asked to name any side effect(s) of which they were aware; investigators (SHH, AD) later grouped similar answers together. To assess utilization of family planning, men were asked if they had ever used anything or tried in any way to delay or avoid getting their partner pregnant (yes/no). If yes, they were asked what method they first used and where they obtained this method (response options: clinic, health centre, hospital, drug shop, or other). They were also asked whether it cost them any money (yes/no) and if yes, how much. In addition, they were asked who was involved in choosing this method (response options: individually, with a partner, or with a health provider). Finally, they were asked if they were currently using anything to delay or avoid getting their partner pregnant (yes/no). Please see questionnaire attached as Additional file 1.

Ethical approval for the study was granted by the Biomedical Science Higher Degrees Research and Ethics Committee, Makerere University; with a research number SBS 354. Additional approvals were obtained from Nakaseke district administration and the Uganda National Council on Science and Technology. All participants gave written informed consent to participate in the study.

## Results

One hundred and seventy-eight men completed the survey. Demographic characteristics are presented in Table 1. The average age was 34 years old (range 18–71). About half (100/178, 56%) had a primary school education or less and most were either Anglican (69/178, 39%) or Roman Catholic (50/178, 29%). Similar percentages of respondents were single (61/178, 34%), cohabitating (61/178, 34%) or married (56/178, 31%) and reported an average of 5 children to date.

**Table 1:** Characteristics of men living in Nakaseke District in rural central Uganda who completed the survey (*N* = 178)

Characteristic	Mean ± SD or n (%)
Years of age	34.1 ± 10.9 (range 18–71)
Education level ≤ primary school	100 (56)
Religion	
Anglican	69 (39)
Roman Catholic	50 (28)
Pentecostal	31 (17)
Muslim	21 (12)
Traditional	7 (4)
Marital status	
Single	61 (34)
Cohabitating	61 (34)
Married	56 (31)
Number of children (*n* = 172)	5.1 ± 4.5

Nearly all of the men surveyed (175/178, 98%) reported some knowledge of family planning. The majority (130/178, 73%) had heard about family planning on the radio, while 43% (77/178) heard about it from health workers, and less than 10% had heard from TV (15/178, 8%), billboards (7/178, 4%) or friends/relatives (5/178, 3%). Nearly all men (162/178, 91%) said they knew of at least one family planning method and all but two could specify at least one method in a free text answer. The most common method named by these participants was the male condom (105/160, 66%), with the next most common being injectable hormonal contraception (87/160, 54%) and birth control pills (83/160, 52%). Some men also named implants, IUDs and female condoms (all 28/160, 16%). The remaining six methods named (vasectomy, tubal ligation, lactational amenorrhea, moon beads, emergency contraception, periodic abstinence) were reported by < 10% of participants (≤ 15/160).

About half of the men (87/178, 49%) reported that they have heard about any side effects of some family planning methods and most (69/87, 79%) offered one or more specific side effects when prompted (Table 2). Men were able to accurately report many of the more common side effects of contraceptive methods, including abnormal uterine bleeding (27/69, 39%) and vaginal dryness (10/69, 14%), while reports of false side effects, such as increased risk of infertility (8/69, 12%), cancer (6/69, 9%), and birth defects/abnormal pregnancies (5/69, 7%), were relatively rare. All other side effects were reported by ≤5% of men.

**Table 2:** Specific side effects reported by a subset of men who reported they had heard of any side effects of some family planning methods (*n* = 69)

Reported side effect	N (%)
Abnormal uterine bleeding	27 (39)
Abdominal pain	12 (17)
Headache/dizziness	11 (16)
Vaginal dryness	10 (14)
Infertility	8 (12)
Decreased libido	7 (10)
Cancer	6 (9)
Birth defects or abnormal pregnancy	5 (7)
Weight loss	4 (6)

Side effects reported by < 5% of men are not included

About half of the men (94/178, 53%) reported they had ever used something or tried in some way to delay or avoid getting their partner pregnant. When asked what method they first used, all but two of these men reported using male condoms, while one of them reported using vasectomy and the other reported using both male condoms and vasectomy. Nearly half (39/94, 41%) received the condoms from a hospital, while smaller numbers obtained condoms from a drug shop (27/94, 29%), a clinic (19/94, 20%), or a health center (9/94, 10%). About half (51/92, 55%) said it cost them money (median cost Uganda Shilling [UGX] 500 (United States Dollar [USD] 0.14), range 300–4000 (USD 0.08–1.12). More than three-quarters (70/93, 77%) said they were the only one involved in choosing this method while a quarter (22/91, 24%) said their partner was involved, and only one man (1/91, 1%) said a health provider was involved. Among these 94 men who reported having ever used contraception, more than three-quarters (71/94, 76%) said they were currently using something to delay or avoid getting their partner pregnant; this translates to 40% of the entire sample (71/178).

## Discussion

The present study quantified knowledge and use of contraception among men living in a rural area in central Uganda. Nearly all men had heard of family planning. Relatively few had heard about family planning from a healthcare worker. This is not surprising: while women commonly access healthcare facilities for antenatal care and childhood immunization visits, men are far less likely to have healthcare needs that bring them to hospitals and clinics where they might encounter accurate family planning information. In contrast, most men reported hearing about family planning from the radio. There have been some efforts to use media (radio, television, print ads, etc.) to increase participation in family planning decision-making and use that have had positive results [14] including in Uganda [15]. A more recent examination of programs in Nigeria, Kenya, and Senegal that included exposure to family planning messages via mass media, print media, interpersonal communication, and community events also found some evidence that exposure to radio advertisements/programs increased men’s reported use of modern contraception. Interestingly, results also suggested that men who attended community events (e.g., community theater) and heard religious leaders speak favorably about family planning were also more likely to report use of modern contraception, two additional approaches that may be effective in areas like Nakaseke [16].

With regard to side effects of contraception, many men could name specific side effects, and the side effects noted were consistent with those reported by men in qualitative studies [9–11]. Reassuringly, false side effects like increased risk of infertility, cancer, and birth defects were reported by a small minority of men and the more commonly reported side effects, like irregular bleeding and vaginal dryness, were accurate. While it is commendable that men report correct rather than incorrect side effects, there is still much work to be done. For example, results from qualitative studies suggest that some men express false corollary beliefs about the ramifications of true side effects. For example, in one qualitative study, men reported that abnormal uterine bleeding led to fatigue and lack of interest in sex among women using contraception, which in turn motivated them to pursue sexual relations with other women [9]. Given that more than two thirds of the married women in Uganda report that contraceptive decision-making is either undertaken jointly or exclusively by male partners [6], education efforts should continue to build the foundation of accurate knowledge about contraceptive side effects among men while also looking for ways to promote greater understanding about the ramifications of such side effects.

Perhaps not surprisingly, men were most familiar with male condoms, but many also named injectable hormonal contraception and birth control pills. There was little familiarity with the most effective contraceptive methods, especially long-acting reversible methods like IUDs and implants. The fact that men are partially or completely responsible for the contraceptive decisions made by the majority of married Ugandan women [6] underscores the central role that men play in family planning decisions in this culture and suggest that increasing knowledge and acceptability of these methods among men could be one avenue for increasing use of these very effective methods. Importantly, such efforts will only be successful if there are also parallel efforts to address lack of availability of these devices and the skilled staff needed to insert and remove them.

This study has limitations. The sample is relatively small, cross-sectional and utilized a convenience sample. Additionally, the flow of the yes/no questions on the survey may have inadvertently limited the number of men who were asked certain questions (e.g., about barriers to family planning). It should also be noted that near universal knowledge of family planning in this survey represents only a general awareness of family planning. It does not necessarily represent specific knowledge of particular family planning methods or an understanding of proper use nor does it speak to an understanding of comparative effectiveness of different methods.

## Conclusions

Despite some limitations, the results of the present study extend those of recent qualitative studies and provide information about the types of family planning information men are lacking and avenues for getting this information to them. An opportunity lies in providing men with accurate family planning information, especially about highly effective methods. As men are not accessing the hospital or clinics often or getting their information from healthcare workers, the venue for such education should include settings outside of healthcare facilities, but staffed with trained healthcare workers.

## Additional file

Additional file 1: Questionnaire for contraceptive use in Nakaseke community survey. Data collection tool. (DOCX 28 kb)

## Abbreviations

ACCESS: African Community Center for Social Sustainability
IUD: intrauterine device
UDHS: Uganda Demographic and Health Survey
UGX: Uganda Shilling
USD: United States Dollar

## References

[1] Ahmed S, Li Q, Liu L, Tsui AO (2012). Maternal deaths averted by contraceptive use: an analysis of 172 countries. Lancet.

[2] Bhutta ZA, Das JK, Bahl R, Lawn JE, Salam RA, Paul VK (2014). Can available interventions end preventable deaths in mothers, newborn babies, and stillbirths, and at what cost?. Lancet.

[3] Rutstein S, Winter R. Contraception needed to avoid high-fertility-risk births, and maternal and child deaths that would be averted. 2015. https://dhsprogram.com/pubs/pdf/AS50/AS50.pdf. Accessed 23 Mar 2018.

[4] United Nations, Department of Economic and Social Affairs, Population Division. Trends in Contraceptive Use Worldwide 2015. 2015. http://www.un.org/en/development/desa/population/publications/pdf/family/trendsContraceptiveUse2015Report.pdf. Accessed 23 Mar 2018.

[5] Ministry of Health, Uganda. Uganda Family Planning Costed Implementation Plan, 2015–2020. Kampala: Ministry of Health, Uganda. 2014 https://www.healthpolicyproject.com/ns/docs/CIP_Uganda.pdf. Accessed 19 Apr 2018.

[6] Uganda Bureau of Statistics (UBOS) and ICF (2018). Uganda Demographic and Health Survey 2016.

[7] Nalwadda G, Mirembe F, Byamugisha J, Faxelid E (2010). Persistent high fertility in Uganda: young people recount obstacles and enabling factors to use of contraceptives. BMC Public Health.

[8] Ouma S, Turyasima M, Acca H, Nabbale F, Obita KO, Rama M (2015). Obstacles to family planning use among rural women in Atiak health center IV, Amuru District, northern Uganda. East Afr Med J.

[9] Kabagenyi A, Jennings L, Reid A, Nalwadda G, Ntozi J, Atuyambe L (2014). Barriers to male involvement in contraceptive uptake and reproductive health services: a qualitative study of men and women’s perceptions in two rural districts in Uganda. Reprod Health.

[10] Sileo KM, Wanyenze RK, Lule H, Kiene SM (2017). “That would be good but most men are afraid of coming to the clinic”: men and women’s perspectives on strategies to increase male involvement in women’s reproductive health services in rural Uganda. J Health Psychol.

[11] Thummalachetty N, Mathur S, Mullinax M, DeCosta K, Nakyanjo N, Lutalo T (2017). Contraceptive knowledge, perceptions, and concerns among men in Uganda. BMC Public Health.

[12] Guttmacher Institute. Contraception and unintended pregnancy in Uganda. 2017. https://www.guttmacher.org/sites/default/files/factsheet/fb-contraception-and-unintended-pregnancy-in-uganda.pdf. Accessed 23 Mar 2018.

[13] Paul B, Ayo AS, Ayiga N (2015). Rural-urban contraceptive use in Uganda: evidence from UDHS 2011. J Hum Ecol.

[14] Mwaikambo L, Speizer IS, Schurmann A, Morgan G, Fikree F (2011). What works in family planning interventions: a systematic review. Stud Fam Plan.

[15] Gupta N, Katende C, Bessinger R (2003). Associations of mass media exposure with family planning attitudes and practices in Uganda. Stud Fam Plan.

[16] Okigbo CC, Speizer IS, Corroon M, Gueye A (2015). Exposure to family planning messages and modern contraceptive use among men in urban Kenya, Nigeria, and Senegal: a cross-sectional survey. Reprod Health.

