# Supplementary material for: Knowledge and use of family planning among men in rural Uganda
Source: BMC Public Health. 2018 Nov 26;18:1294. doi: 10.1186/s12889-018-6173-3 (PMC6258500; doi:10.1186/s12889-018-6173-3)
Supplement: Supplementary file 1 — Questionnaire for contraceptive use in Nakaseke community survey. Data collection tool. (DOCX 28 kb) [file 12889_2018_6173_MOESM1_ESM.docx]

**Questionnaire for Contraceptive Use in Nakaseke Community survey**

**Family planning practices in Nakaseke district, Uganda**

**Principal investigator: Dr. Robert Kalyesubula Mobile Tel: 0772442700/0782348560**

**Study Number Reg. Number Age**

1. **Sub county:** **1.Nakaseke TC** **2.Semuto TC**  **3.Kiwoko TC**  **4.Kasangombe**  **5.Kitto**
2. **Marital status:**  **1.Married** **2.Single**  **3.Cohabiting**  **4.Other specify**
3. **Education level:**  **1.Not educated**  **1.Primary** **2.Secondary** **3.University** **4Tertiary**
4. **Religion:** **1.Roman catholic** **2. Anglican**  **3. Pentecostal**  **4. Muslim** **5.Traditional**
5. **Parity (Number of children for wife/spouse)**
6. **Have you heard about family planning before?**  **Yes** **No if yes, where did you hear it from?**

…………………………………………………………….

1. **Do you know of any family planning method?** **Yes**. No **If yes, which one(s) do you know?**

**a……………………………………………..**

**b……………………………………………..**

**c……………………………………………..**

**d……………………………………………..**

**e………………………………………………**

**f……………………………………………….**

**g……………………………………………….**

1. **Have you heard about any side effect of some family planning methods?**  **Yes** **No if yes, which one(s) have you heard about?**

**i…………………………………………………….**

**ii……………………………………………………**

**iii…………………………………………………..**

**iv……………………………………………………**

**v…………………………………………………….**

**vi……………………………………………………**

1. **Have you ever used anything or tried in any way to delay or avoid getting your wife pregnant?** **Yes**  **No If yes, which way?**

**i…………………………………………………….**

**ii……………………………………………………**

**iii…………………………………………………..**

**iv……………………………………………………**

**v…………………………………………………….**

**vi……………………………………………………**

1. **Where did you get this service from?** **1. Clinic** **2.Health centre** **3. Hospital**

**4.drug shop others specify…………………………………………**

1. **Did it cost you any money?**  **Yes** **No**

**If yes, how much in Uganda shillings?.................................................**

1. **How did you choose your family planning method?**  **1-individually**  **2-with a partner,**  **3-with a health provider**
2. **Are you currently using anything to delay or avoid getting your spouse/partner pregnant?**

**Yes** **No If No, reason for stopping …………………………**

1. **If yes, would you want to space your children, delay or avoid getting pregnant?**  **Yes** **No If Yes, what is limiting you?..............................................................................**
2. **Has your spouse/partner had an unintended pregnancy in the past?**  **Yes** **No**
